# Study protocol for the design, implementation, and evaluation of the STRATIFY clinical decision support tool for emergency department disposition of patients with heart failure

**DOI:** 10.1186/s43058-025-00779-w

**Published:** 2025-10-17

**Authors:** Sunil Kripalani, Deonni P. Stolldorf, Anna L. Sachs, Jennifer B. Barrett, Shilo H. Anders, Laurie L. Novak, Dandan Liu, Joseph Miller, Bory Kea, Isaac Schlotterbeck, Alan B. Storrow

**Affiliations:** 1 Section of Hospital Medicine, Department of Medicine, Vanderbilt University Medical Center, Nashville, TN USA; 2 Center for Health Services Research, Vanderbilt University Medical Center, 2525 West End Avenue, Suite 1200, Nashville, TN USA; 3 School of Nursing, Vanderbilt University, Nashville, TN USA; 4 Department of Anesthesiology, Vanderbilt University Medical Center, Nashville, TN USA; 5 Center for Research and Innovation in System Safety, Vanderbilt University Medical Center, Nashville, TN USA; 6 Department of Biomedical Informatics, Vanderbilt University Medical Center, Nashville, TN USA; 7 Department of Biostatistics, Vanderbilt University Medical Center, Nashville, TN USA; 8 Henry Ford Health and Michigan State University Health Sciences, Detroit, MI USA; 9 Center for Policy and Research in Emergency Medicine, Department of Emergency Medicine, Oregon Health & Sciences University, Portland, OR USA; 10 Department of Emergency Medicine, Vanderbilt University Medical Center, Nashville, TN USA

**Keywords:** Heart failure, Emergency department, Risk prediction, Clinical decision support

## Abstract

**Background:**

In the emergency department (ED), clinicians often make challenging, high-pressure decisions within a short time frame. Clinical decision support (CDS) tools integrated into the electronic health record can provide evidence-based support. Yet, numerous implementation barriers limit the broad use of such tools in ED settings. CDS tools could be particularly helpful for patients presenting to the ED with an acute exacerbation of heart failure (AHF), a common and costly medical condition for which patients are typically admitted to the hospital. We developed and implemented STRATIFY, a validated risk prediction model that effectively identifies AHF patients at low risk of 30-day adverse events who could potentially be discharged home from the ED.

**Methods:**

This article describes a multi-center study to 1) develop a stakeholder-informed CDS-based implementation process for STRATIFY, 2) use novel statistical methods to overcome data integration challenges to the real-world implementation of predictive models in the ED, and 3) evaluate the implementation and effectiveness of the newly developed STRATIFY CDS at 7 EDs to guide decision-making to admit or discharge patients with AHF. The study’s multi-level implementation strategy is tailored to each site and informed by site assessments (including pre-visit surveys, on-site ED visits, and virtual interviews), small group discussions with patients and caregivers, and iterative user-centered design to develop and refine the STRATIFY CDS. Overcoming data challenges for real-time predictive models involves accommodating missing risk factor data while still generating valid predictions of risk. In the evaluation of effectiveness, we will evaluate ED disposition (admit/discharge) for patients with AHF, as well as potential adverse outcomes, using an interrupted time-series design at 7 participating EDs. The study will evaluate implementation outcomes ranging from acceptability to sustainability using electronic health record data and surveys of clinicians and patients.

**Discussion:**

This study uses a stakeholder-informed, iterative design approach to develop a tailored CDS-based process supported by a multi-level implementation strategy to incorporate a validated risk prediction tool into the care of patients with AHF in the ED. The study will advance methods to close the evidence-practice gap in the care of emergency department patients.

**Supplementary Information:**

The online version contains supplementary material available at 10.1186/s43058-025-00779-w.

Contributions to the Literature

•Protocol for multi-level implementation and evaluation of clinical decision support (CDS) in the understudied emergency department setting
•Use of rigorous, stakeholder-informed, iterative user-centered design to develop an acceptable CDS tool feasible to deploy in the busy, real-world environment
•Application of Consolidated Framework for Implementation Research (CFIR) and Expert Recommendations for Implementing Change (ERIC) implementation strategies to enhance rigor and reproducibility and inform future implementation research in the emergency department

## Background

Emergency department (ED) clinicians are on the front lines of care for patients with unplanned medical needs. They must often make challenging, high-pressure decisions within a condensed time frame [1]. The use of clinical decision support (CDS) risk prediction tools may lessen these burdens, allowing ED clinicians to make evidence-based decisions quickly, and such tools are perceived favorably when they are aligned with clinical workflows [2]. The use of electronic health record (EHR) systems has enhanced the feasibility and potential usability of CDS tools to improve health care delivery [3]. Integrating CDS tools into the EHR may improve the standardization and quality of care in the ED [4–6]. However, EHR-integrated CDS is still not broadly used in ED settings.

Previous research suggests two overarching factors impede CDS implementation. First, the usefulness of CDS in the ED has been hindered by limitations in the timely and uniform availability of structured EHR-based data that would allow the delivery of CDS to the ED clinician within a suitable timeframe [7, 8]. The second factor is the insufficient involvement of clinicians, patients, and organizational leaders in determining tool features, user interfaces, and forms of deployment [9]; active stakeholder engagement is crucial for understanding clinical needs, improving CDS usability, and integrating CDS seamlessly into workflows [7, 10–13].

In this study, we engaged diverse EDs with varying levels of health information technology (IT) capacity, practice patterns, and organizational characteristics in the development and evaluation of a CDS-based implementation strategy for an existing validated risk prediction tool. As a test case, we chose to focus on the critical clinical domain of acute heart failure (AHF), a common and costly medical condition treated in the ED. Over 80% of ED patients with AHF are admitted to the hospital, but as many as 50% of these patients could be safely discharged home [14, 15]. Hospitalization and readmissions account for much of the $39.2 billion in estimated US annual healthcare expenditures for heart failure [16]. Identifying even a small additional percentage of ED patients with AHF who could be safely discharged would avoid potentially unnecessary hospitalizations and save significant healthcare resources [17, 18].

STRATIFY, a risk prediction model that identifies low-risk patients with AHF who could be safely discharged from the ED, was motivated by the urgent need for improvement in this clinical area. Developed and externally validated by our team [19–21], STRATIFY uses data available in the first 3 h of an ED evaluation to readily identify patients with AHF at low risk of 30-day death or serious complications. The prediction model calculates a score using 13 risk factors—8 collected at triage, 1 from electrocardiogram, and 4 from initial blood testing [19]. Integrating STRATIFY within the EHR for real-time automated use at the patient level will give clinicians the right information at the right time to inform the disposition (i.e., whether to admit or discharge) of patients with AHF presenting to the ED; however, there is a translational gap for practice implementation, which this study addresses.

## Objective

**Fig. 1:**
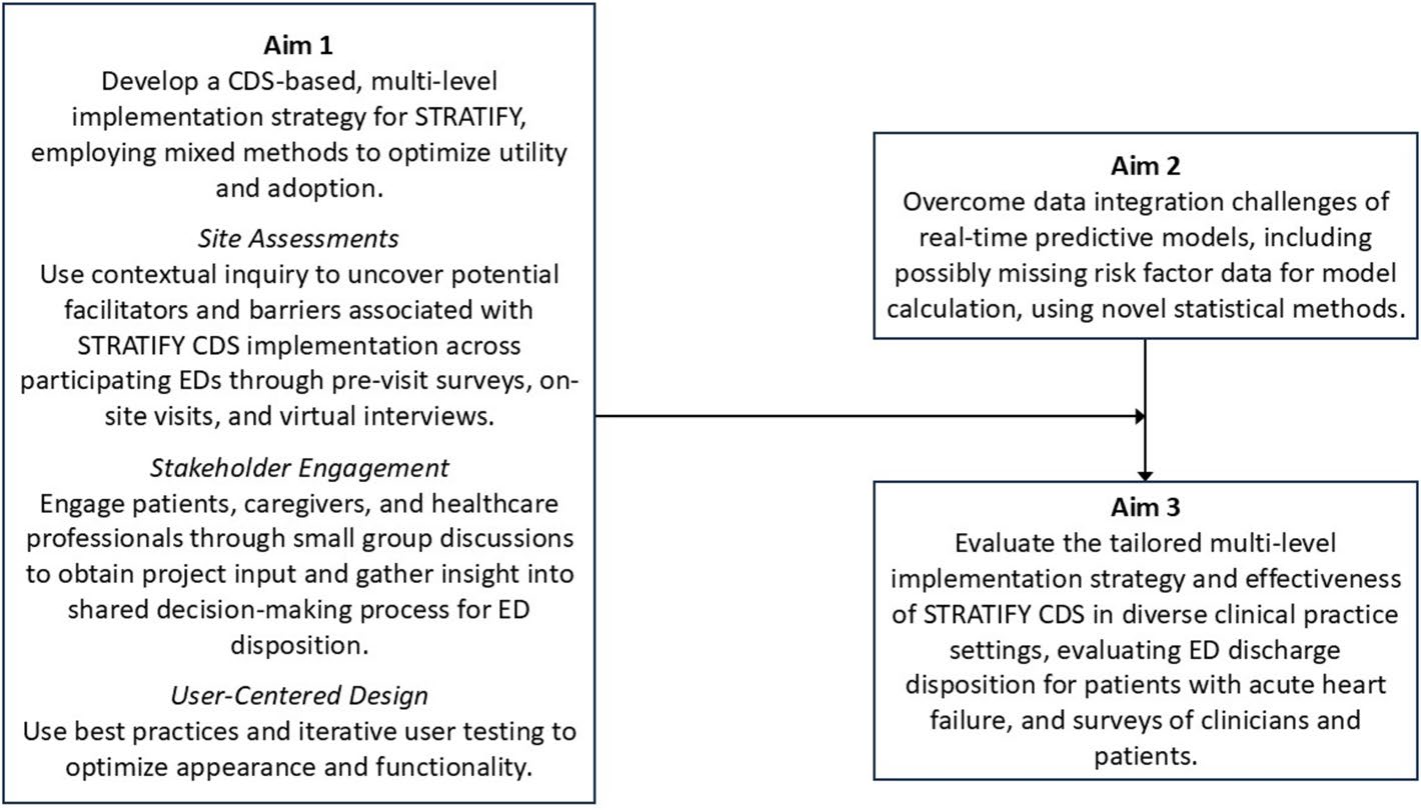
Summary schema of study

This study aims to 1) develop a stakeholder-informed, CDS-based implementation process for STATIFY (Aim 1), 2) overcome data integration challenges to the real-world implementation of predictive models in the ED using novel statistical methods (Aim 2), and 3) evaluate the implementation and effectiveness of the newly developed STRATIFY CDS (Aim 3) (Fig. 1). Our long-term goal is to develop a scalable and shareable process and generalized strategy for CDS implementation in EDs as a vehicle to improve the efficiency and quality of emergency care.

This article describes the overall methods for this study, including a summary of the approach for Aims 1 and 2, and a more extensive description of the ongoing evaluation of the effectiveness and implementation of the STRATIFY CDS (Aim 3).

## Theoretical framework

We used the Consolidated Framework for Implementation Research (CFIR) and the Expert Recommendations for Implementing Change (ERIC) taxonomy of implementation strategies to develop our implementation process for STRATIFY [22, 23]. We also use the recommendations of Proctor and colleagues for specifying and reporting implementation strategies (i.e., name and define each strategy and specify the actor, action, action target, temporality, dose, implementation outcome affected, and justification) to report on our implementation strategies to enhance replicability [24]. Using these frameworks, coupled with the principles of user-centered design and a stakeholder-informed multi-level implementation strategy, enhances rigor while bridging the translational gap that hinders CDS implementation in the ED.

## Overall study design and methods

The overall study uses an interrupted time series design to evaluate the effect of STRATIFY CDS implementation on the ED disposition of patients with AHF at multiple EDs. Our main hypothesis is that STRATIFY CDS implementation will increase the home discharge rate, safely reducing admissions for low-risk patients with AHF, without evidence of an increase in ED revisits or unscheduled hospital admission for AHF at 5 days, or in several 30-day patient safety outcomes used in the initial model validation (detailed below).

### Study sites and participants

The study sites are 7 EDs across 3 geographically diverse sites: Vanderbilt University Medical Center (VUMC, 1 ED), Oregon Health & Science University (OHSU) and its partner (2 EDs), and Henry Ford Health (HFH, 4 EDs). Each site is implementing STRATIFY CDS as standard of care across the EDs. For Aim 1, we used a combination of purposive and snowball sampling to identify participants from different stakeholder groups across sites for development and planning implementation. The stakeholder groups included patients, hospital administrators, health IT, ED physicians, and ED nurses. For the biostatistical modeling (Aim 2) and evaluation of effectiveness and implementation (Aim 3), the study uses EHR data on patients presenting to the ED with AHF across the study sites. This study was approved by the institutional review boards at VUMC, OHSU, and HFH.

## Approach

### Aim 1: develop a CDS-based implementation process for STRATIFY, drawing on the rigorous engagement of multiple types of stakeholders from each site

This aim included three distinct phases: site assessment and contextual inquiry, patient and caregiver focus groups, and user-centered design of the CDS tool and complementary implementation strategies [25–28]. We developed an implementation blueprint to organize these pre-implementation activities, specifying a timeline as well as the details of each activity.

Site assessments were carried out by a team that included a nurse implementation scientist, a health systems anthropologist, an ED physician, a physician health services researcher, and a clinical informatician. The team used contextual inquiry methods to uncover potential facilitators and barriers associated with STRATIFY CDS implementation across participating EDs through pre-visit surveys, on-site ED visits, and virtual interviews [29]. The pre-visit surveys assessed the implementation climate, organizational readiness for change, and attitudes of physicians and nurses toward using CDS for decision-making in ED disposition decisions. During site visits, the team observed and discussed workflows related to heart failure assessment, EHR use and data availability, and decision-making around specific interventions and disposition. Interviews engaged 18 stakeholders (6 at VUMC, 8 at OHSU, and 4 at HFHS), who represented hospital administration, health IT, ED physicians, and ED nurses. Interview coding was guided by the CFIR and enabled a better understanding of the internal setting, people, process, and technology, and considerations for implementation. Heterogeneity between sites in levels of health IT capacity, practice patterns, and other organizational characteristics provided diverse input for a more robust process and helped identify potentially necessary adaptations for implementation at each site.

Next, we conducted small group discussions with patients and caregivers to gather insight into shared decision-making during the ED disposition process and obtain other project input. These were done using Community Engagement Studios, a structured roundtable session held virtually [30].

We then used an iterative user-centered design approach to develop and refine the STRATIFY CDS to optimize its utility and adoption [31, 32]. Specifically, this third step involved development of the tool’s interface using established usability principles, as well as conducting 30-min usability evaluations with 18 physicians and nurses across the participating EDs using the cognitive walkthrough method and finishing with the system usability scale. The usability participants considered one to three case scenarios and responded to questions gauging how the presentation of the STRATIFY risk prediction score would contribute to their decision-making. We documented issues identified by participants regarding the STRATIFY interface, pathway of access (e.g., “clicks” required), and score display. We iterated the design and modified it after our initial evaluation with six users. The usability testing results were summarized and shared with each site, along with recommendations for site-specific interface revisions. The usability of the revised interfaces and workflows were retested and found to meet usability objectives, including no safety-related design issues.

As noted above, an implementation blueprint organized these activities, which were carried out over a 20-month period. The CDS was the central component of a multi-component, multi-level implementation strategy. Using input received from stakeholders in each phase, the team specified additional components of this implementation strategy. The full set of strategies is presented in Appendix Table 1. Pre-implementation activities included assessing readiness and identifying barriers and facilitators, identifying, and preparing champions, and developing educational materials. The implementation phase activities (carried out in Aim 3 below) included facilitating the relay of clinical data to clinicians, developing, and implementing tools for quality monitoring, and purposely reexamining implementation. For each strategy, we specified the actor, action, action target, temporality, dose, mechanism, and implementation outcome affected [24].

### Aim 2: overcoming data integration challenges the real-world implementation of predictive models in CDS using novel statistical methods

The key challenges addressed in Aim 2 relate to the possibility of missing risk factor data for model calculation. In this real-world setting, treating clinicians may not always order all of the tests which comprise the model parameters, or results may be delayed, yet model output is needed within the first few hours of patients’ presentation to the ED to inform clinical decision-making. The statistical approach is described in depth in a separate article [33] and summarized here. We developed a novel statistical method to obtain sub-models for all possible scenarios of incompleteness. Each sub-model aims to calculate a risk score for a new patient with a certain scenario of incomplete STRATIFY risk factors. We conducted comprehensive simulation studies to compare our sub-model approach with alternative approaches (i.e., the one-step-sweep sub-model approach and the imputation by fixed chained equations approach) [34].

We then validated and recalibrated the original STRATIFY model and applied the proposed sub-model approach using retrospective and de-identified EHR data, with varied time periods across sites to obtain as much data as possible for model validation; the EHR extraction period began on January 1, 2018, for all sites, with end dates of February 6, 2023 (VUMC), May 26, 2023 (HFHS), and February 16, 2024 (OHSU). The data included patient demographic and clinical characteristics, the 13 risk factors included in STRATIFY and their timestamps of first availability in the EHR, and 30-day STRATIFY patient safety outcomes from all 3 sites for patients presenting to participating EDs with a diagnosis of AHF (identified by ICD-10 code). The analyses evaluated the completeness and plausibility of all STRATIFY risk factors to understand potential data challenges in implementation at each site [35]. We assessed model performance for each sub-model and chose the sub-models with performance comparable to that of the full model for implementation. This ensures that we only use models that can provide reliable risk calculations in the presence of incompleteness. The results from this Aim will help us to strategically deliver reliable risk prediction results in the 7 participating EDs.

### Aim 3: evaluate the implementation and effectiveness of the newly developed STRATIFY CDS

Among patients with AHF presenting to the participating EDs, Aim 3 will evaluate the effect of implementing the STRATIFY CDS using a tailored multi-component strategy (Appendix Table 1) on ED discharge disposition. This Aim will also examine implementation outcomes ranging from acceptability to sustainability, guided by Proctor’s Implementation Outcomes Framework [36, 37]. Conducting the project in EDs with different EHR installations and organizational characteristics will illuminate factors affecting both implementation and effectiveness in diverse real-world settings.

#### Design

The *effectiveness* evaluation will use an interrupted time series design, drawing on 4 years of data (2-year retrospective data collected before implementation, a brief run-in period, and 2-year prospective data collected during implementation). To evaluate *implementation,* we will conduct an observational study and use surveys of clinicians (ED physicians and advanced practice providers), patient surveys, site data, and EHR data.

#### STRATIFY CDS implementation and implementation support

Implementation of the STRATIFY CDS will be led by a central team for consistency, in collaboration with a clinical champion from each site, following the context specific, tailored multilevel strategy developed in Aim 1. The preparatory work in Aim 1, including the performance of local consensus discussions, should contribute to the establishment of a supportive implementation climate at each site, including leader engagement, clear communication of the goals of the STATIFY CDS, and the provision of appropriate resources to assist clinicians in using the CDS and in communicating with patients and families about its role in their care [38]. Similar implementation strategies have been shown to successfully increase safe discharge in patients with low-risk chest pain and pulmonary embolus [39]. With these preparations, we planned a sequential roll-out over a total 6-month period, staggered across sites. The educational aspects of the implementation will utilize internal ED communication networks. The clinical staff will be educated about the STRATIFY CDS through peer networks, meetings, and flyers posted in physical locations and distributed through email. The STRATIFY CDS will be made available to all clinicians involved in the ED disposition process, and use of the tool will be voluntary.

#### Evaluation of effectiveness

##### Study population

The patient population for analysis is the same as the population eligible for the intervention: adults with known chronic heart failure (defined by past medical history/problem list or ICD-10 code) who have a natriuretic peptide ordered as part of ED care or are administered an intravenous diuretic in the ED, as indicators of acute exacerbation. Clinical documentation, ED diagnoses, or billing codes for AHF are not completed in real-time, so cannot be used for the immediate identification of AHF patients. For the effectiveness study, based on historical patient volumes, we expect to use EHR data from approximately 7,725 patients meeting these inclusion criteria.

##### Outcome measures

The following data will be extracted from the EHR at each site: patient demographic and clinical characteristics; STRATIFY risk variables, score, and disposition recommendations; patient discharge disposition; and 5-day and 30-day clinical outcomes. The primary outcome for effectiveness is ED disposition, defined as home discharge (either immediately from the ED or after a brief [< 24-h] stay in an outpatient observation unit based in the ED) vs. hospitalization. Brief observation before home discharge is common in outpatient AHF care. The safety outcomes are 5-day ED revisit or unscheduled hospital admission for AHF (ICD-10: I50.xx or I11.0) and the 30-day adverse events used in the initial STRATIFY development and validation studies (i.e., death, cardiopulmonary resuscitation, mechanical cardiac support, intubation or mechanical ventilation, emergent dialysis, percutaneous coronary intervention, coronary artery bypass grafting, and acute coronary syndrome) [19]. We hypothesize that STRATIFY CDS implementation will increase the home discharge rate without evidence of an increase in 5-day or 30-day safety outcome rates.

##### Analysis

We will conduct segmented regression analysis to compare changes in the monthly rate of home discharge and in 5-day and 30-day safety outcomes across the 2-year baseline period and the 2-year implementation period, excluding a washout period when the CDS goes live at a given site (T0). Segmented regression analysis allows the estimation of differences in outcomes due to an abrupt change in practice pattern (i.e., STRATIFY CDS implementation). Both immediate changes (i.e., comparing intercepts) and changes over time (i.e., comparing slopes) are examined. We will include random effects for EDs to account for heterogeneity across EDs and correlations within EDs. The statistical power for these analyses depends on factors including distribution of data points before and after the intervention, data variability, effect strength, and the presence of time-dependent confounders (e.g., seasonality), which will be evaluated prior to analysis [40].

##### Power consideration

To estimate power, we ran simulations following an interrupted time series design and estimated immediate (intercept) and temporal (slope) changes in the primary outcome of home discharge. Assuming a 20% baseline home discharge rate and a modest correlation (*r* = 0.20) between pre- and post- outcomes over 48 time points (monthly over 4 years), with a Type I error rate of 0.05, the estimated sample size of *N* = 643 per month (i.e., 7,725 patients with AHF annually) will have a power of 80% to detect an odds ratio of 1.2, which is equivalent to a 3% absolute increase in the home discharge rate. This change represents a meaningful clinical difference and would result in substantial resource savings.

#### Evaluation of implementation

To evaluate implementation outcomes, we will track site activities; extract information from the EHR on CDS use; survey a sample of ED clinicians; and incorporate a post-discharge survey of patients with AHF.

##### Study sample

For clinician surveys, we will establish an advisory panel of ED clinicians from each health system (approximately 20 in total) who agree to be surveyed periodically. They will be administered brief web-based surveys through REDCap, beginning at implementation go-live and up to two additional times during the implementation period of up to 24 months. For patient surveys, a sample of 70 patients (10 per participating ED) will be asked to complete a 5-min survey on their satisfaction with discharge decision-making.

##### Outcome measures

We will assess the implementation outcomes of acceptability, adoption, appropriateness, feasibility, fidelity, implementation cost, penetration, and sustainability (Table 1). Perhaps the most important implementation outcome in this setting is penetration, or the degree to which the STRATIFY CDS is integrated into practice. To evaluate penetration, we will use EHR audit log data gathered continuously at each site to provide information on which clinicians use the CDS and for what percentage of eligible patients. Clinician surveys will include self-report measures and open-ended questions to assess STRATIFY CDS use in clinical decision-making.

**Table 1:** STRATIFY clinical decision support (CDS) implementation outcomes to be assessed

Outcome	Source	Details	Timing*
**Acceptability**	Clinician survey	Ratings of clinicians finding STRATIFY more or less acceptable	T0, T1, T2
	Patient survey	Satisfaction with STRATIFY CDS-guided discharge disposition	Within 6 months of discharge
**Adoption**	Site data	Decision by site to implement STRATIFY CDS	T0
**Appropriateness**	Clinician survey	Did score influence disposition decision-making? Why or why not? Did patients receive the report, and was it used for shared decision-making? In what situations was STRATIFY CDS most helpful, and why? In what situations was STRATIFY CDS least helpful, and why?	T1, T2
**Feasibility**	EHR	% of AHF encounters for which the score was successfully calculated at any point while in the emergency department % of AHF encounters where STRATIFY score was available the last time the physician tried to get a score % of STRATIFY encounters where score was successfully calculated despite missing data % of STRATIFY-scored encounters where STRATIFY scores were recalculated	Monitored throughout implementation
	Clinician survey	What factors (workflow, competing demands) influenced feasibility?	T1, T2
**Fidelity**	Site data	Extent to which each site carried out the implementation strategy as planned	T1
**Implementation Cost**	Site data	Cost of technical time to program and maintain CDS Cost of other implementation components (training materials, staff time, etc.)	End of implementation
**Penetration**	EHR	Did the clinician have a patient with AHF? For what % of these patients did they access the tool?	Monitored throughout implementation
	Clinician survey	For approximately what % of patients with acute heart failure did the clinician look up the STRATIFY CDS tool? For approximately what % of patients with AHF did the clinician use the tool for shared decision-making with the patient?	T1, T2
**Sustainability**	Site data	Above measures of acceptability, appropriateness, feasibility, and penetration in year 2 of implementation	Maintenance period

* T0 = start of implementation, T1 = several months into implementation, T2 = approximately12 months into implementation

##### Analysis

The data will be tabulated and summarized using descriptive statistics and scale scores for validated measures. We will summarize the implementation outcomes for each ED separately and for each participating site (combining data across EDs per site). For clinician-based measures extracted from the EHR (e.g., feasibility, penetration), we will also evaluate whether values differ according to characteristics such as age, number of years in practice, and clinical time (e.g., average number of clinical shifts per month). For open-ended questions, the research team will conduct rapid qualitative analysis to identify key themes. We will document relevant feedback and retain it for future reference and for dissemination to the community of researchers and analysts working on the implementation of predictive tools.

## Discussion

This study uses a stakeholder-informed, iterative design approach to develop a tailored CDS-based process supported by a multi-level implementation strategy to incorporate a validated risk prediction tool into the care of patients with AHF in the ED. This study focuses on a context (the ED) and impactful clinical area (AHF and cardiovascular disease more generally) that are both known to have relatively low use of decision-support tools [10]. Our approach ensures meaningful engagement across broad stakeholder groups (patients, hospital administration, health system and ED settings). The study additionally addresses the feasibility challenges of CDS implementation in the ED, where immediate clinical decisions may need to be made with incomplete information, through novel statistical approaches for dealing with missing data. A unique aspect of this study is its focus on identifying patients at low risk of complications and using the information to dynamically bend the curve in hospital admissions. Finally, this study will contribute to the implementation of CDS tools in ED settings through a systematic and rigorous evaluation of the effectiveness and implementation of this CDS.

One potential benefit of this study is a better understanding of how to appropriately tailor the implementation of the STRATIFY risk prediction tool to local contexts to facilitate adoption and implementation by individuals in ED settings. Our stakeholder-engaged approach will also generate critical insight on the dissemination, implementation, and sustainability of risk prediction tools for other conditions. Our study will contribute to altering the current conservative paradigm in the ED and hospital evaluation and management of patients with AHF and is the next step toward achieving our larger objective of more appropriate allocation of hospital resources to reduce costs and improve health care delivery without adversely affecting patient outcomes. We intend for the CDS process and systematic implementation strategy developed in this study to be scalable and shareable across diverse EDs in a variety of clinical domains.

This systematic implementation-effectiveness evaluation in diverse EDs will also contribute to the broader implementation science literature involving implementation facilitators and barriers in busy and complex clinical settings, an area lacking robust evidence [8, 41]. Specifically, we expect to fill important knowledge gaps related to 1) the implementation strategies that are most effective in ED settings and for CDS implementation, 2) the effective adaptation of strategies to the local context to enhance implementation and clinical outcomes, and 3) the mechanisms (i.e., processes by which strategies work to deliver the desired benefits) of implementation strategies that facilitate positive implementation outcomes.

## Supplementary Information

Supplementary Material 1.

## Data Availability

Data sharing is not applicable to this article as no study datasets have yet been generated or analyzed.

## Abbreviations

AHF: Acute heart failure
CDS: Clinical decision support
CFIR: Consolidated Framework for Implementation Research
ED: Emergency department
EHR: Electronic health record
ERIC: Expert Recommendations for Implementing Change
HFH: Henry Ford Health
IT: Information technology
OHSU: Oregon Health & Science University
VUMC: Vanderbilt University Medical Center
