# Supplementary material for: Study protocol for the design, implementation, and evaluation of the STRATIFY clinical decision support tool for emergency department disposition of patients with heart failure
Source: Implement Sci Commun. 2025 Oct 17;6:107. doi: 10.1186/s43058-025-00779-w (PMC12535060; doi:10.1186/s43058-025-00779-w)
Supplement: Supplementary file 1 — Supplementary Material 1. [file 43058_2025_779_MOESM1_ESM.docx]

Appendix Table 1

| **Implementation Strategy** | **Definition** | **Specification of Strategy** | | | | | **Mechanism/Justification** | **Implementation Outcome Affected** |
| --- | --- | --- | --- | --- | --- | --- | --- | --- |
|  |  | **Actor** | **Action** | **Action Target** | **Temporality** | **Dose** |  |  |
| Access new funding | Access new or existing money to facilitate the implementation | PIs | STRATIFY PIs apply for NIH funding to support project development and implementation | NIH | Pre-implementation | Initial submission, revision | Additional funding is needed to support development of user-centered interface, implementation, and evaluation |  |
| Inform local opinion leaders | Inform clinicians identified by colleagues as opinion leaders or “educationally influential” about the clinical innovation in the hopes that they will influence colleagues to adopt it | Contact PI, site PIs | Identification of advisory committee members at each site.  We worked with department chairs and clinical leads to get approval for the CDS.  Storrow reached out to residency director and chief resident to get their perspectives and help with dissemination of the tool. | Clinicians, nurses, administrators | Pre-implementation | Initial outreach, 1-2 rounds of snowball sampling | Engagement of local opinion leaders is critical for buy-in and influencing local adoption | Acceptability, Penetration |
| Assess for readiness and identify barriers and facilitators | Assess various aspects of an organization to determine its degree of readiness to implement, barriers that may impede implementation, and strengths that can be used in the implementation effort | Site visit teams, qualitative team | Administer surveys on organizational climate and readiness  interview local health care professionals at different sites  (Include summary of site visit interviews and surveys)  Sites (7)  OHSU – DW  OHSU – HMC  HFHS – Main  HFHS – Fairlane  HFHS – Wyandotte  HFHS – Bloomfield  VUMC | Clinicians, nurses, administrators | Pre-implementation | Baseline surveys and interviews conducted | Mixed-methods assessment of readiness, barriers, and facilitators guides development of CDS features and displays, as well as implementation strategies to align with local needs and norms  (How do observation units and other lesser levels of care fit in the implementation, i.e., some sites may have additional lesser care options / more comfort with them) |  |
| Promote network weaving | Identify and build on existing high-quality working relationships and networks within and outside the organization, organizational units, team, etc. to promote information sharing, collaborative problem solving, and a shared vision/goal related to implementing the innovation | Site PIs, clinicians | Ensure we know and get involved with the other existing QI work going on at these sites to integrate into practice more seamlessly  We worked with the user-centered design team + Health IT developers and managers, who have experience working with other initiatives. | Clinicians, administrators, quality improvement champions/leaders | Pre-implementation, at the sign-off stage | Semi-regularly | Integrating with pre-existing quality improvement efforts and the systems used to support them at the local level can help drive adoption and sustainability. | Reach, adoption, sustainability |
| Conduct local consensus discussions | Include local clinicians and other stakeholders in discussions that address whether the chosen problem is important and whether the clinical innovation to address it is appropriate | User-centered design team | Engaged clinician users in decisions about STRATIFY score thresholds, data presentation, and design features | Clinicians | Pre-implementation, design phase | Baseline discussions | Engaging front-line users facilitates buy-in around decisions that must be made regarding STRATIFY score calculation, data presentation, and CDS features | Acceptability, appropriateness |
| Identify and prepare champions | Identify and prepare individuals who dedicate themselves to supporting, marketing, and driving through an implementation, overcoming indifference or resistance that the intervention may provoke in an organization | MPIs, site PIs | We identified champions and PI explained the study to them in one-on-one conversations. | Clinicians, nurses | Pre-implementation, end of design phase, before study launch | Once for each site (~ 15 minutes per conversation) | Providing centralized training to site champions who will then lead education at each site provides some consistency while also engaging local assets | Reach, adoption |
| Promote adaptability | Identify the ways a clinical innovation can be tailored to meet local needs and clarify which elements of the innovation must be maintained to preserve fidelity | MPIs, research team | Team meetings to discuss ongoing implementation and determine if any contextual changes necessitate adaptations.  Example: VUMC had to develop and validate a new model when the troponin lab test changed. | Implementation team members at each site | Implementation phase | Ongoing | Team discussions and reflections on implementation processes and outcomes | Reach, adoption |
| Develop educational materials | Develop and format manuals, toolkits, and other supporting materials in ways that make it easier for stakeholders to learn about the innovation and for clinicians to learn how to deliver the clinical innovation | Nurse scientist and graphic artist for flyers; contact PI for email text and webinar | Contact PI shared two slide decks during faculty meetings on multiple occasions to introduce the tool and screenshots.  Created video overviews of STRATIFY, including specific screenshots for each site. | Clinicians, nurses, patients | Pre-implementation, design phase | Once | Flyers/ webinar/ emails will increase staff knowledge and understanding, buy-in, and subsequent adoption | Reach, adoption |
| Obtain and use patient and family feedback | Develop strategies to increase patient and family feedback on the implementation effort | Nurse scientist, contact PI | Obtain input on patient-facing educational materials  Presented slides to the Patient Family Advisory Council. | Patients | Pre-implementation, design phase; implementation phase | Once pre-implementation; ongoing during implementation | Patient and/or family input will help shape messaging around use of a score in disposition decision-making; patient perspectives will inform future implementation and scale-up | Appropriateness, feasibility |
| Tailor strategies | Tailor the implementation strategies to address barriers and leverage facilitators that were identified through earlier data collection | Implementation scientist PI, nurse scientist, site PIs | Customize the emphasis on certain strategies, modes, or duration of training based on pre-implementation assessment, additional site input, and site-specific clinical resources and care delivery patterns. | Clinicians, nurses | Pre-implementation, ongoing during implementation | Once for planning purposes before study start, ongoing | Different care options at the sites (e.g., observation unit, hospital at home program, heart failure clinics) could impact willingness to use STRATIFY. Tailoring strategies and messaging to each site’s needs and norms will optimize education, uptake, and practice change. | Appropriateness, reach, adoption |
| Distribute educational materials | Distribute educational materials (including guidelines, manuals, and toolkits) in person, by mail, and/or electronically | Site PIs | Department of Emergency Medicine website has a page dedicated to STRATIFY, which is linked in the EHR if clinicians click on the learn more link.  Info about STRATIFY shared in department newsletter and in department-wide email.  Patient flyers available in the EHR for dissemination during a visit. | Clinicians, nurses, patients | Pre-implementation, quarterly during implementation | Once before study start, once per quarter ongoing | Distribution of informational materials will increase staff knowledge and understanding, buy-in, and subsequent adoption | Reach, adoption |
| Conduct educational meetings (group) | Hold meetings targeted toward different stakeholder groups (e.g., faculty, residents, administrators, other organizational stakeholders) to teach them about the clinical innovation | Contact PI, site PIs | Give a brief in-service at each site with each group of stakeholders about STRATIFY, ideally incorporated into existing departmental meetings. | Clinicians, nurses, administrators | Pre-implementation, throughout implementation | Once at study start, as needed to reach additional groups | Group education is an efficient way to brief key stakeholders on the project and provide opportunity for questions | Reach, adoption |
| Conduct educational outreach visits (individual) | Have a trained person meet with clinicians in their practice settings to provide education on the clinical innovation with the intent of changing the clinician’s practice | Site PIs | We had planned to reach out to clinicians who did not participate in the general meeting to brief them on the CDS tool, but this was deemed unfeasible. | Clinicians | Pre-implementation, early during implementation | Once per clinician | Individual outreach to clinicians is worthwhile to ensure education about the project, when group education did not occur | Reach, adoption |
| Facilitate relay of clinical data (STRATIFY scores) to clinicians | Provide as close to real-time data as possible about key measures of process/outcomes using integrated channels of communication in a way that promotes use of the targeted innovation | Biostatistics and clinical informatics teams, via CDS tool | Integrate STRATIFY risk scores into clinical workflow for every eligible patient using CDS tool, using either native Epic or FHIR (depending on site).  Developed a data pre-fetching process to quicken calculation and provision of score. | Clinicians, nurses | Throughout implementation | Available in background during patient encounter, escalated at pre-specified time in evaluation (~4 hrs, or when key labs return) | Bringing the risk stratification score into clinical workflow is a fundamental part of implementation | Reach, adoption |
| Develop and implement tools for quality monitoring | Develop, test, and introduce into quality-monitoring systems the right input – the appropriate language, protocols, algorithms, standards, and measures (of processes, patient outcomes, and implementation outcomes) that are often specific to the innovation being implemented | Biostatistics team, Implementation team, site PIs | Extract STRATIFY CDS usage rates and summarize at the clinician and site levels; extract ED disposition rates for eligible patients and summarize at the clinician and site levels.  Add balancing measure of outcomes (mortality, ED recidivism, safety events).  Creation of clinician survey to be disseminated to advisory committee members.  Developed monitoring dashboard in Tableau. | Clinicians, administrators  (Determine at the site level if admin should be involved with the monthly data quality monitoring outcomes) | Throughout implementation; overall summary upon completion | Once at start of implementation, available throughout | Monitoring usage of CDS and changes in ED disposition rates in real-time enables practice feedback and identifies the need for booster training or adjustment of approach if needed | Reach, adoption, sustainability |
| Develop and organize quality monitoring systems | Develop and organize systems and procedures that monitor clinical processes and/or outcomes for the purpose of quality assurance and improvement | Informatics personnel, implementation team | Set up indicators to check that STRATIFY is working as intended (e.g., triggered for right patients, flowsheets working accurately, data pre-fetch working correctly, monitor for algorithmic drift) | Clinical data | Pre-implementation, Throughout implementation | Once at start of implementation, available throughout | Verify fidelity of steps and algorithmic calculations | Fidelity, sustainability |
| Audit and provide feedback | Collect and summarize clinical performance data over a specified time period and give it to clinicians to monitor, evaluate, and modify behavior | Biostatistics team, site PIs | Using the tools for quality monitoring, prepare and deliver reports for practice-level or clinician-level feedback  Using Tableau usage reports to inform conversations about adaptations.  Ensure buy-in from clinicians. | Clinicians | Throughout implementation | Once monthly | Regular feedback to participating clinician groups fosters engagement, and allows discussion of progress as well as opportunities for improvement | Reach, adoption, sustainability |
| Purposely reexamine the implementation | Monitor progress and adjust clinical practices and implementation strategies to continuously improve the quality of care | PIs, site PIs | Assess progress using the monitoring tools, and provide booster training or adjunctive implementation strategies.  PIs provided additional training when changes were made to CDS.  Sampling of cases to see how/if the risk score aligned with clinical assessment and the score influenced their decision. | Clinicians, nurses, patients | 2-3 pre-specified points during implementation (e.g., at 6, 12, and 18 months) | Once per pre-specified time point | Monitoring and taking steps to boost the success of implementation is appropriate in real-world intervention studies | Reach, adoption, sustainability |
